# UN peacekeeper health and risk factors --- a systematic scoping review

**DOI:** 10.1186/s41256-024-00351-4

**Published:** 2024-04-10

**Authors:** Quan Yuan, Yong Chen, Shili Liu, Qingning Huang, Miaomiao Liao, Jiani Zhou, Zhaogang Li, Ying Li

**Affiliations:** Department of Social Medicine and Health Service Management, Army Medical University (Third Military Medical University), Chongqing, China

**Keywords:** United Nations peacekeepers, Health status, Health risk factors, Health protection, Global health, Scoping review

## Abstract

**Background:**

Conflicts, natural disasters, and complex emergencies present substantial health challenges to United Nations (UN) peacekeepers deployed in mission areas. This scoping review aims at summarizing previous research on the health of UN peacekeepers and identifies issues for further investigation.

**Methods:**

Following the Preferred Reporting Items for Systematic Reviews and Meta-Analyses (PRISMA) extension for Scoping Reviews, we systematically searched Web of Science, PubMed, EMBASE, Scopus and China National Knowledge Infrastructure (CNKI) for English and Chinese literature published from April 1997 to November 2023. A data charting form was developed by two reviewers to extract relevant themes and provided narrative descriptions.

**Results:**

We screened 1079 de-duplicated records and included 143 studies in this scoping review. There were 112 studies on the health status of UN peacekeepers, with more than half on mental health problems such as stress and anxiety. Many studies explored the health status of UN peacekeepers in African countries deployed from mainly U.S., Canada, U.K., China, Australia and Norway. There were 39 studies on the health risk factors of UN peacekeepers, including natural environmental, social environmental, psychological, behavioral lifestyle, biological factors and health service factors. There were 62 articles on the health protection of UN peacekeepers, mainly based on previous deployment experience, with a lack of theoretical guidance from global health perspectives. This scoping review found that health problems of UN peacekeepers are complicated, and whose impacts are cross-border. Social environmental factors were explored the most among health risk factors. Disease prevention measures, medical and health measures, and psychosocial measures were the main health protection for UN peacekeepers.

**Conclusions:**

This scoping review highlighted that health problems of UN peacekeepers were typical global health issues with complicated and cross-border health risk factors. Therefore, comprehensive strategies could be taken from global health perspectives, including multi-phases (before-deployment, during-deployment, and post-deployment), multi-disciplines (public health, medicine, politics, health diplomacy, and others), and multi-levels (the UN, host countries, troop-contributing countries, the UN peacekeeping team, and UN peacekeepers).

**Supplementary Information:**

The online version contains supplementary material available at 10.1186/s41256-024-00351-4.

## Introduction

Since the World War II, the promotion of peace and stability has been a key priority outlined in the Charter of the United Nations (UN), calling for collective action from the international community [1]. The first UN peacekeeping mission was established in May 1948, when the UN Security Council authorized the deployment of military observers to the Middle East. Over the past 70 years, more than 1 million peacekeepers have served in over 70 UN peacekeeping operations [2, 3]. At present, over 100,000 military, police, and civilian personnel from 125 countries serve in 14 active peacekeeping operations. For example, in response to the crisis in South Sudan, the Security Council reinforced the United Nations Mission in South Sudan and reprioritized its mandate towards the protection of civilians, the delivery of humanitarian aid, as well as the implementation of the Cessation of Hostilities Agreement [4]. UN peacekeeping has been recognized as a unique and dynamic instrument for assisting conflict-affected countries to achieve lasting peace.

Since 1988, more than 4100 UN peacekeepers have sacrificed their lives in missions, of which more than half occurring in the current peacekeeping missions [1]. Between 2000 and 2017, 2042 peacekeepers lost their lives, of which 879 peacekeepers died because of diseases (43.0%), surpassing those because of incidents (29.5%) and violence (19.9%) [3]. UN peacekeepers face complex health risk factors, including poor environmental sanitation, imbalanced diet, limited social network, and unhealthy behaviors such as alcohol and drug abuse. Previous research has mentioned that UN peacekeepers had the frequent occurrence of mental health problems such as anxiety and depression, and the high incidence of infectious diseases such as malaria and Acquired Immune Deficiency Syndrome (AIDS), as well as other conditions such as skin diseases and insomnia [5]. UN peacekeepers have also been associated with the disease transmission to broader population, such as the cholera outbreak in Haiti in 2010 and the transmission of Human Immunodeficiency Virus (HIV) during and after deployment [6]. The UN has previously adopted resolutions emphasizing the health protection of UN peacekeepers and broader population they engage with, such as their families and colleagues [7]. For instance, Resolution 1308 (2000) recognized the need to incorporate HIV/AIDS prevention awareness and skills training for UN peacekeepers, and Resolution 2668 (2022) stressed the importance of mental health and psychosocial support for both UN peacekeepers and people they interact with [8, 9]. Moreover, the UN has provided peacekeepers with the Core Pre-deployment Training Materials on health knowledge, such as personal hygiene measures and disease prevention guidelines [10].

To the best of our knowledge, there is no comprehensive scoping review on collecting peer-reviewed literature on the health of peacekeepers. Therefore, the aim of this review is to map the existing literature on the health problems, risk factors, and protective measures of UN peacekeepers, whilst identifying the health of UN peacekeepers is a typical global health issue and strategies should be taken from global health perspectives to protect their health.

## Methods

### Search strategy

This scoping review adhered to the Preferred Reporting Items for Systematic Reviews and Meta-Analyses (PRISMA) extension for Scoping Reviews [11]. The framework proposed by Arksey and O’Malley [12] was used to map the literature pertaining to our research topic. This scoping review identified eligible studies published from April 1997 to November 2023 on health status of UN peacekeepers, health risk factors of UN peacekeepers and health protection of UN peacekeepers. Four major databases including Scopus, PubMed, EMBASE and China National Knowledge Infrastructure (CNKI) were searched. We used a mixture index terms and free text to maximize the retrieval of potentially relevant studies. The terms of “peacekeeper”, “health”, “disease”, “infection”, “prevalence”, “risk factor”, “protection”, “prevention”, “measure”, “control”, “policy” and “strategy” were used as keywords and text words to search (see Additional file 1). Reference lists of identified manuscripts were hand-searched.

### Inclusion and exclusion criteria

The inclusion criteria included:

1. Type of studies: cross-sectional studies, case-control studies, cohort studies, retrospective studies, longitudinal studies, and medical records report.
2. Research subjects: UN peacekeepers.
3. Outcome variables:

(i) Health status of UN peacekeepers: mental health problems, infectious diseases, and other health problems;
(ii) Health risk factors of UN peacekeepers: natural environmental factors, social environmental factors, psychological factors, behavioral lifestyle factors, biological factors, and health service factors;
(iii) Health protection of UN peacekeepers: disease prevention measures, medical and health measures, psychological measures, and diplomatic measures.

The exclusion criteria were: (1) articles not specifically related to the health of UN peacekeepers; (2) articles with no research design (opinion pieces, blogs, reviews, news articles, conference paper and gray literature); (3) articles without full texts;

If there were multiple reports of the same study, only the article with a full report was included.

### Study selection

Two reviewers identified studies using the inclusion and exclusion criteria. Each reviewer screened the titles and abstracts of identified studies independently to preliminarily assess their eligibility according to the inclusion/exclusion criteria. All reviewers made the decision to include/exclude a study by discussion and consensus where there were disagreements regarding the eligibility of studies. Each of the selected full-text papers was read thoroughly, several times by two reviewers to capture all relevant information and to ensure that nothing important was missed.

### Data extraction

Two reviewers independently read the full texts of all initially selected manuscripts and finally included eligible articles according to the inclusion/exclusion criteria. Differences were resolved by discussion and consensus among reviewers. We established an Excel form with a reference number assigned for each included article, and then extracted following data from each study: health status, health risk factors, health protection, author/year, WHO regions: troop-contributing countries, host countries, study designs, and main outcomes. Each extracted data was classified and summarized on outcome variables, and was recorded in the Excel form. Any disagreement was resolved through discussion and consensus among all reviewers Tables 1, 2, and 3.

**Table 1:** Studies on health status of UN peacekeepers

Health Status	Diseases	Author/year	WHO regions: Troop-contributing countries	Host countries	Study designs	Main outcomes
Mental Health	PTSD	Ward et al. 1997 [13], Mehlum et al. 1999 [14], Bramsen et al. 2000 [15], Brarmsen et al. 2001 [16], Mehlum et al. 2002 [17], Asmundson et al. 2003 [18], Hotopf et al. 2003 [19], Forbes, 2005 [20], Bolton et al. 2006 [21], Litz et al. 2006 [22], Greenberg et al. 2008 [23], Souza et al. 2008 [24], Thoresen et al. 2008 [25], Richardson et al. 2008 [26], Richardson et al. 2009 [27], Rademaker et al. 2009 [28], Maguen et al. 2009 [29], Connorton et al. 2011 [30], Fernando et al. 2011 [31], Engdahl et al. 2011 [32], Richardson et al. 2012 [33], Giorgi et al. 2017 [34], Dixit et al. 2018 [35], Elrond et al. 2019 [36], Gjerstad et al. 2020 [37]	Western Pacific Region: Australia	Vietnam, Somalia	Medical report, Questionnaire	High PTSD scores, Substantial proportion of PTSD veterans.
			Region of the Americas: U.S., Canada, Brazil	Somalia, Haiti Multination	Longitudinal study, Cohort study, Questionnaire, Longitudinal study	PTSD prevalence at Time 1 (8.2%) and at Time 2 (10.2%), PTSD severity not related to cardiovascular problems, PCL-M screening criteria for PTSD (73.6%), Veterans with PTSD, Negative affect traits on PTSS.
			European Region: Norway, U.K., Netherlands, Denmark	South Lebanon, Lebanon, Multination, Bosnia, Yugoslavia, Cambodia, Afghanistan	Questionnaire, Cross-sectional study, Epidemiological study, Control groups, Cohort study, Interview	PTSD among Norwegian peacekeepers (5.2%), Service-related stress and PTSD, Watching drama relevant to their experiences, PTSD prevalence from 3.6 to 5.5%, Exposure to traumatic events contributed to PTSD, No increase in the number of PTSD, PTSD among formerly deployed soldiers.
			South-East Asia Region: India, Multination	Multination, Kosovo	Cross-sectional study, Retrospective study, Questionnaire, Sample study, Interview	No cases of PTSD, Veterans who screened positive for PTSD scored higher, PTSD symptoms’ impact upon chronic pain experiences, PTSD predicted by level of anxiety and depression, PTSD symptom models and factor structure, Combat related PTSD, PTSD symptom and trauma history.
	Stress	Ballone et al. 2000 [38], Wessely et al. 2006 [39], Nikolova et al. 2007 [40], Adler et al. 2008 [41], Britt et al. 2011 [42], Barnes et al. 2013 [43], Souza et al. 2015 [44], Mustafa et al. 2021 [45]	Region of the Americas: U.S.	Multination, Kosovo	Questionnaire	Stressful experience, Routine exposures cause high levels of stress, Incident stress debriefing.
			European Region: Italy, Bulgaria, U.K.	Bosnia, Kosovo	Cross-sectional study, Longitudinal study, Questionnaire	High stress level during deployment, Determination and characteristics of stress, Perceived stress from professional difficulties and frustrations.
			Eastern Mediterranean Region: Pakistan	Democratic Republic of Congo	Cross-sectional study	Differences between the married and unmarried peacekeepers on stress.
			Multination	Multination	Questionnaire, Interview	Acute psychological stress.
	Anxiety and depression	Orsillo et al. 1998 [46], Ippolito et al. 2005 [47], Wessely et al. 2006 [39], Sareen et al. 2007 [48], Jia et al. 2009 [49], Chen et al. 2010 [50], Zhu et al. 2010 [51], Zhang et al. 2010 [52], Shao et al. 2011 [53], Li et al. 2012 [54], Chen et al. 2012 [55], Chen et al. 2012 [56], Li et al. 2012 [57], Li et al. 2012 [58], Selič et al. 2013 [59], Zhang et al. 2013 [60], Sun et al. 2014 [61], Dixit et al. 2018 [35], Zhao et al. 2019 [62], Gjerstad et al. 2020 [63]	European Region: U.K., Norway, Slovenia	Bosnia, Lebanon, Kosovo	Questionnaire, Cross-sectional study	Perceived stress came from professional difficulties and frustrations.
			Region of the Americas: Canada	Haiti	Cross-sectional study	Generalized anxiety disorder.
			South-East Asia Region, Eastern Mediterranean Region: Pakistan, Bangladesh	Liberia	Questionnaire	Peacekeepers in two countries had different scores in mental health problems such as anxiety and depression.
			South-East Asia Region: India	Multination	Cross-sectional study	Increased incidence of anxiety and depression.
			Western Pacific Region: China	The Democratic Republic of the Congo, Nigeria, Sudan, Liberia, Lebanon, Namibia, Ghana, Multination	Questionnaire	The Chinese version of the Chinese Military Mental Health Scale was used to test peacekeepers’ mental status including anxiety and depression levels.
			Multination	Kosovo, Somalia	Questionnaire	Generalized anxiety disorder, depression.
	Psychiatric disorders	Ward et al. 1997 [13], Orsillo et al. 1998 [46], Richardson et al. 2012 [33]	Region of the Americas: Canada	Multination	Questionnaire	Self-reported psychiatric illness and suicidal ideation among peacekeepers with PTSD.
			Western Pacific Region: Australia	Somalia	Questionnaire	One-fifth of Australian soldiers who served in Somalia had significant. Levels of psychiatric morbidity
			Multination	Somalia	Questionnaire	Over one third of participants met criteria for psychiatric caseness.
Infectious diseases	HIV/AIDS	Hu et al. 2005 [64], Ross et al. 2006 [65], Guo et al. 2006 [66], Jin et al. 2008 [67], Lowicki-Zucca et al. 2009 [68], Assan et al. 2021 [69]	Western Pacific Region: China	The Democratic Republic of Congo, Liberia, Sudan	Medical report, Questionnaire	Reported AIDS cases among Chinese peacekeepers.
			Region of the Americas: U.S.	sub-Saharan Africa	Content analyses	The exposure, vulnerability, and impact of HIV on peacekeepers.
			African Region: Nigeria	West Africa	Questionnaire	Situationally focused individual HIV intervention
			Multination	Multination	Prevalence comparison	HIV prevalence of the peacekeeping mission is higher than that of the host country.
	Malaria and cholera	Hu et al. 2005 [64], Jeremy et al. 2005 [70], Jin et al. 2008 [67], Xie et al. 2009 [71], Wang et al. 2011 [72], Wei et al. 2011 [73], Ma et al. 2011 [74], Sun et al. 2012 [75], Juliao et al. 2013 [76], Kapoor et al. 2013 [77], Lewnard et al. 2015 [78], Fernando et al. 2016 [79], He et al. 2017 [80], Kunkel et al. 2017 [81], Fernando et al. 2017 [79], Qi et al. 2018 [82], Guerra et al. 2019 [83], Li et al. 2021 [84]	Western Pacific Region: China	Sudan, South Sudan, Liberia, The Democratic Republic of Congo	Medical report, Retrospective study, Questionnaire	The provision of care for patients with malaria and cholera.
			South-East Asia Region: Sri Lanka, Nepal	The Central African Republic, Haiti, Multination	Questionnaire, Interviews, Stochastic modeling	30.8% of peacekeepers (37/120) had 44 symptomatic episodes of malaria during deployment.
			European Region: U.K.	Multination	Case study	High rates of transmission of malaria.
			Region of the Americas: Peru, Guatemala	The Central African Republic, The Democratic Republic of the Congo	Case study	The first imported malaria cases represent in Peru, Identifying malaria cases and risk factors for malaria acquisition.
	HEV and HAV		Eastern Mediterranean Region: Egypt	The Democratic Republic of Congo	Case report	Spontaneous rupture of spleen with complicated falciparum malaria.
			Multination	Haiti	Stochastic model	Cholera importation and transmission.
		Drabick et al. 1997 [85], Gambel et al. 1998 [86], Li et al. 2019 [87]	South-East Asia Region: Bangladesh	Haiti	Epidemiological study	High genomic identity with Asian strains of HEV and dissimilarity with the Mexican strain.
			Western Pacific Region: China	South Sudan	Case study	A life-threatening case of HAV infection.
			Multination	Haiti	Questionnaire	Pakistan (62%), India (37%), Nepal (37%), Bangladesh (27%), Djibouti (13%), Honduras (6%), Guatemala (5%), Haiti (3%).
	Other infectious diseases	Gambel et al. 1999 [88], Jia et al. 2018 [89], ter Meulen et al. 2001 [90], Zhao et al. 2015 [91]	Western Pacific Region: China	Africa, Liberia	Clinical study, Medical report	A live-attenuated YFV vaccine strain, EVD outbreak in West Africa.
			Multination	Haiti, Sierra Leone, Australia, Central Africa	Interview, Medical report, Case study, Cohort study	Dengue, Lassa fever, Dengue, Tungiasis.
Other health problems		Adams et al. 1997 [92], Thoresen et al. 2003 [93], Hu et al. 2005 [64], Hébert et al. 2007 [94], Li et al. 2008 [95], Xie et al. 2009 [71], Sun et al. 2011 [96], Korzeniewski et al. 2011 [97], Tian et al. 2011 [98], Glad et al. 2012 [99], Sun et al. 2014 [100], Yang et al. 2014 [101], Strand et al. 2014 [102], Bolognesi et al. 2016 [103], Zhou et al. 2016 [104], Strand et al. 2016 [105], Enkhtsetseg et al. 2016 [106], Tang et al. 2017 [107], He et al. 2017 [80], Chen et al. 2018 [108], Qi et al. 2018 [82], Zhao et al. 2018 [109],Wang et al. 2018 [110], Bullman et al. 2019 [111], Qi et al. 2019 [112], Zuo et al. 2020 [113], Liu et al. 2021 [114] Korzeniewski, 2011 [115] Okulate, 2004 [116] Duarte, 2015 [117]	Western Pacific Region: China	Mali, Sudan, Lebanon, Liberia, South Sudan, The Democratic Republic of Congo	Expert consultation, Retrospective study	Respiratory diseases, Fracture, Intermittent diarrhea, Eczema, Insomnia, Mosquito bites, Trauma, Low back and leg pain, Oral ulcers, Injuries, Skin and soft tissue defects.
	Oral, skin, gastrointestinal, musculoskeletal, respiratory, urinary, ophthalmic, otolaryngologic and gynecological diseases, leukemia		Region of the Americas: Canada, U.S., Brazil	Bosnia, Kosovo, Haiti	Case study, Cohort study, Medical report, Interview	Musculoskeletal injuries, Mortality (leukemia, respiratory disease, respiratory cancer, and heart disease).
			European Region: Sweden, Multination, Italy, Norway, U.K., Poland	Afghanistan, Middle East, Iraq, Kosovo, Lebanon, Bosnia, Central Africa	Questionnaire, Medical report, Cohort study	Food and water-borne diseases, Leukemia, Fatal Accidents, Rubella, Non-battle injuries.
			African Region: Nigeria	Yugoslavia and Liberia	Interview	Homicidal violence.

**Table 2:** Studies on health risk factors of UN peacekeepers

Health risk factors	Author/year	WHO regions: Troop-contributing countries	Host countries	Study designs	Main outcomes
Natural environmental factors	Hu et al. 2005 [64], Lehtom¨aki et al. 2005 [118], Jin et al. 2008 [67], Korzeniewski et al. 2011 [97], Chen et al. 2012 [119], Liang et al. 2014 [120], Zhao et al. 2019 [62], Liu et al. 2021 [114]	Western Pacific Region: China	The Democratic Republic of Congo, Sudan	Medical report, Questionnaire, Retrospective control study	Natural environmental factors (harsh natural environment, poor geographical environment, hot climate, abundant rainfall).
		European Region: Finland	Kosovo	Questionnaire	Natural environmental factors (mold, fungi, dust).
		Multination	The Middle East	Medical report	Natural environmental factors (heat, wind, sand, dust, local fauna, low temperature, and mountain conditions).
Social environmental factors	MacDonald et al. 1998 [121], Ballone et al. 2000 [38], Mehlum et al. 2002 [17], Thoresen et al. 2003 [122], Maguen et al. 2004 [123], Hu et al. 2005 [64], Ippolito et al. 2005 [47], Dirkzwager et al. 2005 [124], Wessely et al. 2006 [39], Jia et al. 2009 [49], Proctor et al. 2009 [125], Zhou et al. 2016 [104], Wang et al. 2018 [110], Dixit et al. 2018 [35], Lamb et al. 2018 [126], Zhao et al. 2019 [62], Silveira-Rodrigues et al. 2021 [127]	Western Pacific Region: China, New Zealand	The Democratic Republic of the Congo, Mali, Lebanon, Multination	Medical report, Questionnaire, Retrospective study	Social environmental factors (turbulent social environment, complex war environment, differences in language and culture, different rules and regulations in the second-level peacekeeping hospital, poor local hygiene conditions, family and job roles).
		European Region: Italy, Norway, Netherland, U.K.	Bosnia, Lebanon, Multination, West Africa	Cross-sectional study, Questionnaire, Cohort study	Social environmental factors (economic conditions, family relationships, unemployment, exposure to dangerous situations, lower level of education, reduced marriage rate, the lack of interoperability).
		South-East Asia Region: India	Multination	Cross-sectional study	Social environmental factors (Family Relationships).
		Region of the Americas: Brazil, U.S.	Haiti, Kosovo, Bosnia	Questionnaire, Cohort study	Social environmental factors (work-to-family enrichment support, family stressors and financial difficulties, differences in support structures).
Psychological factors	Bramsen et al. 2000 [15], Dirkzwager et al. 2005 [124], Ippolito et al. 2005 [47], Maguen et al. 2006 [121], Souza et al. 2008 [24], Jia et al. 2009 [49], Proctor et al. 2009 [125], Shao et al. 2011 [53], Britt et al. 2011 [42], Orme et al. 2014 [128], Silva et al. 2015 [129], Zhou et al. 2016 [104], Lamb et al. 2018 [126], Zhao et al. 2019 [62], Gjerstad et al. 2020 [37]	Western Pacific Region: China, Australia	Timor-Leste or the Solomon Islands, The Democratic Republic of the Congo, Lebanon, Multination	Questionnaire, Retrospective study	Psychological factors (higher hardiness scores are associated with lower levels of reported psychological distress and physical ill-health, resilience, life stress events and emotional experiences, lack of patience, loss of emotional control, longing for family members, instability state of mind).
		European Region: Netherlands, Norway, U.K.	Lebanon, Yugosla, Multination	Cross-sectional study, Questionnaire, Interview	Psychological factors (personal barriers to disclose experiences and current unemployment, vulnerabilities and exposure to traumatic events, confidence and team cohesiveness to build personnel’s resilience).
		Region of the Americas: Brazil, U.S.	Haiti, Multination, Kosovo, Bosnia	Longitudinal study, Questionnaire, Cohort study	Psychological factors (negative affect traits, no associations with CD4 or CD8 T cell numbers, personality hardness, lack of confidence in care and fears of stigmatization).
Behavioral lifestyle factors	Mehlum et al. 1999 [14], Alan et al. 2000 [130], Thoresen et al. 2004 [93], Xie et al. 2009 [71], Proctor et al. 2009 [125], Zhang et al. 2010 [52], Connorton et al. 2011 [30], Zhou et al. 2016 [104], Liu et al. 2021 [114], Qu et al. 2022 [131]	Western Pacific Region: China	Lebanon, Nigeria, Namibia, Ghana, The Democratic Republic of the Congo, Liberia, South Sudan	Retrospective study, Questionnaire, Medical report, Cross-sectional study	Behavioral lifestyle factors (group living, occupational activities and work-related stress, customs and lifestyles, training intensity, intensive labor, performing tasks at high temperatures, coping styles and resilience).
		European Region: Norway	South Lebanon, Multination	Questionnaire, Interview	Behavioral lifestyle factors (alcohol consumption, Misuse of alcohol and other substances, suicide risk factors)
		Region of the Americas: U.S.	Somalia, Bosnia	Questionnaire, Cohort study	Behavioral lifestyle factors (combat and sexual harassment, completing high demands and low control tasks).
		Multination	Kosovo	Questionnaire	Behavioral lifestyle factors (Combat, alone, peacekeeping relief work).
Biological factors	Hu et al. 2005 [64], Shao et al. 2011 [53], Wang et al. 2018 [110]	Western Pacific Region: China	Mali, Lebanon, The Democratic Republic of the Congo	Medical report, Questionnaire	Biological factors (poor sanitary conditions, high prevalence of severe infectious diseases, high resistance to malaria parasites).
Health service factors	Hu et al. 2005 [64], Shao et al. 2011 [53], Wang et al. 2018 [110]	Western Pacific Region: China	Mali, Lebanon, The Democratic Republic of the Congo	Medical report, Questionnaire	Health service factors (difficult collection and follow-up of case data, increasing acute mental health stressors through counseling and protection, anti-malaria medicines and materials).

**Table 3:** Studies on health protection of UN peacekeepers

Health protection	Author/year	WHO regions: Troop-contributing countries	Host countries	Study designs	Main outcomes
Disease prevention measures	Adams et al. 1997 [92], Gambel et al. 1999 [88], Kitchener et al. 2002 [132], Hu et al. 2005 [64], Jeremy et al. 2005 [70], Ross et al. 2006 [65], Thoresen et al. 2006 [133], Guo et al. 2006 [66], Jin et al. 2008 [67], Lowicki-Zucca et al. 2009 [68], Ma et al. 2011 [74], Wei et al. 2011 [73], Juliao et al. 2013 [76], Yang et al. 2014 [101], Lewnard et al. 2015 [78], Fernando et al. 2016 [79], He et al. 2017 [80], Sood et al. 2017 [134], Tang et al. 2017 [107], Bonham et al. 2021 [135], Liu et al. 2021 [114], Li et al. 2021 [84]	Western Pacific Region: China, Australia	Mali, Sudan, South Sudan, The Democratic Republic of the Congo, Lebanon, Liberia	Medical report, Questionnaire, Retrospective study	Disease prevention measures (improving the health prevention system and providing medical examinations for team members, enhancing health education such as AIDS to strengthen prevention awareness, preventing and treating common diseases, improving the health support capacity among peacekeepers, initiating an emergency plan for handling cholera outbreaks, fly eradication, disinfection, managing food and water sources, anti-allergic drugs, anti-malarial drugs, antifungal drugs, skin disease prevention, vaccination, early notification of cases and prevention of transmission through isolation of cases and collaboration).
		Region of the Americas: Guatemala	The Democratic Republic of the Congo	Case study	Disease prevention measures (adherence to chemoprophylaxis).
		South-East Asia Region: Sri Lanka	Multination	Interviews	Disease prevention measures (knowledge on malaria chemoprophylaxis).
		African Region: Nigeria	West Africa	Questionnaire	Disease prevention measures (individual interventions).
		European Region: U.K., Norway	South Sudan, Multination, Bosnia–Herzegovina	Cohort study, Case study	Disease prevention measures (diagnosis and management of infectious diseases, malaria protection policy, firearms control, improved detection systems for mental health problems in the military, immunization).
		Multination	Haiti, Multination, Central Africa	Interview, Prevalence comparison, Stochastic model, Cohort study	Disease prevention measures (enforcement of existing field preventive medicine doctrine, knowledge of HIV and prevention, screening and vaccination, health education and environmental control).
Medical and health measures	Adams et al. 1997 [92], Gambel et al. 1999 [136], Hu et al. 2005 [64], Forbes et al. 2005 [20], Hébert et al. 2007 [94], Li et al. 2008 [95], Maguen et al. 2009 [29], Ray et al. 2009 [137], Wang et al. 2011 [72], Sun et al. 2012 [75], Liu et al. 2012 [138], Yang et al. 2014 [101], Zhao et al. 2015 [91] Fernando et al. 2017 [79], He et al. 2017 [80], Yang et al. 2017 [139], Tang et al. 2017 [107], Xin et al. 2018 [140], Li et al. 2019 [87], Qi et al. 2019 [112], Pei et al. 2020 [141], Liu et al. 2021 [114]	Western Pacific Region: China, Australia	South Sudan, Multination, Sudan, Liberia, Mali, The Democratic Republic of the Congo, Lebanon	Case study, Medical report, Retrospective study	Medical and health measures (vaccines, hospital treatment, specialized PTSD treatment units, active anti-malarial treatment, timely and comprehensive care for patients, isolation measures, psychological care, assess the preparation of personnel and materials, equip specialized professionals and complex equipment, ensuring material supply, optimize the diet structure, diagnosis of infectious diseases and drug preparations, dietary nutrition, preventive medicine, selecting high-quality surgeons, improving linkage mechanism, examination and treatment plan, disinfection of drinking water, anti-malarial treatment, frequent hand washing, preventing the spread of disease, allocation and deployment of health care workers, improving systems and procedures).
		Region of the Americas: Canada	Bosnia, Somalia, Rwanda, Former Yugoslavia	Case study, Interview	Medical and health measures (primary prevention activities and the capacity to provide physiotherapy services, mind-body complementary therapies).
		South-East Asia Region: Sri Lanka	The Central African Republic	Questionnaire	Medical and health measures (better coordination with overseas healthcare services and the establishment of directly observed chemoprophylaxis).
		European Region: U.K.	Bosnia–Herzegovina	Case study	Medical and health measures (effective surveillance and microbiology laboratory support).
		Multination	Kosovo, Haiti	Questionnaire, Interview, Medical report	Medical and health measures (a thorough evaluation of severity of subclusters and an individual profile of symptoms, standardized medical surveillance).
Psychosocial measures	Ward et al. 1997 [13], Mehlum et al. 1999 [14], Ballone et al. 2000 [38], Doody et al. 2002 [142], Thoresen et al. 2003 [122], Okulate et al. 2004 [116], Wessely et al. 2006 [39], Greenberg et al. 2006 [143], Guo et al. 2007 [144], Dyrstad et al. 2007 [145], Nikolova et al. 2007 [40], Jin et al. 2008 [67], Li et al. 2008 [95], Maguen et al. 2009 [29], Fernando et al. 2011 [31], Wong et al. 2011 [146], Chen et al. 2012 [56], Li et al. 2012 [54], Li et al. 2012 [57], Liu et al. 2012 [138], Barnes et al. 2013 [43], Souza et al. 2015 [44], Fu et al. 2017 [147], He et al. 2017 [80], Tang et al. 2017 [107],Dixit et al. 2018 [35], Qi et al. 2018 [82], Saito et al. 2018 [148], Xin et al. 2018 [140], Rodrigues-Silveira et al. 2022 [149]	European Region: U.K., Norway, Italy, Bulgaria, Ireland	Bosnia, South Lebanon, Kosovo, The Mediterranean, Multination	Epidemiological study, Questionnaire, Cross-sectional study, Medical report, Interview	Psychosocial measures (watching TV programs, changing regulations, approaches to stress management, psychosocial support for veterans and their families, gun control, fostering intrinsic motivation toward physical training, physiological stress assessment and screening of mental health risk, effective predeployment resilience building programmes).
		Region of the Americas: Canada, Brazil, U.S.	Bosnia, Haiti, Kosovo	Case control study, Longitudinal study, Questionnaire	Psychosocial measures (training, guidance, organizational affective commitment, work engagement, positive influence of organizational support programs).
		South-East Asia Region: India	Multination	Cross-sectional study	Psychosocial measures (pre-screening, pre-deployment mental health training).
		Western Pacific Region: Australia, China, Japan	Somalia, Sudan, Lebanon, Mali, South Sudan, Liberia	Questionnaire, Retrospective study, Medical report	Psychosocial measures (the selection, training, preparation and debriefing among UN peacekeepers, mental health education through various forms such as lectures and knowledge competitions, psychological counseling, the United Nations medical standard working procedures, the construct validity and reliability of the Tachikawa Resilience Scale, foreign language learning, carrying out recreational activities).
		African Region: Nigeria	Yugoslavia and Liberia	Interview	Psychosocial measures (adequate communication with home could reduce maladjustment).
Diplomatic measures	Guerra et al. 2019 [83], Assan et al. 2021 [69]	Region of the Americas: Peru, U.S.	The Central African Republic, sub-Saharan Africa	Case study, Content analyses	Diplomatic measures (elimination campaigns, HIV-related national policies/legislations).

## Results

### Description of studies

**Fig. 1:**
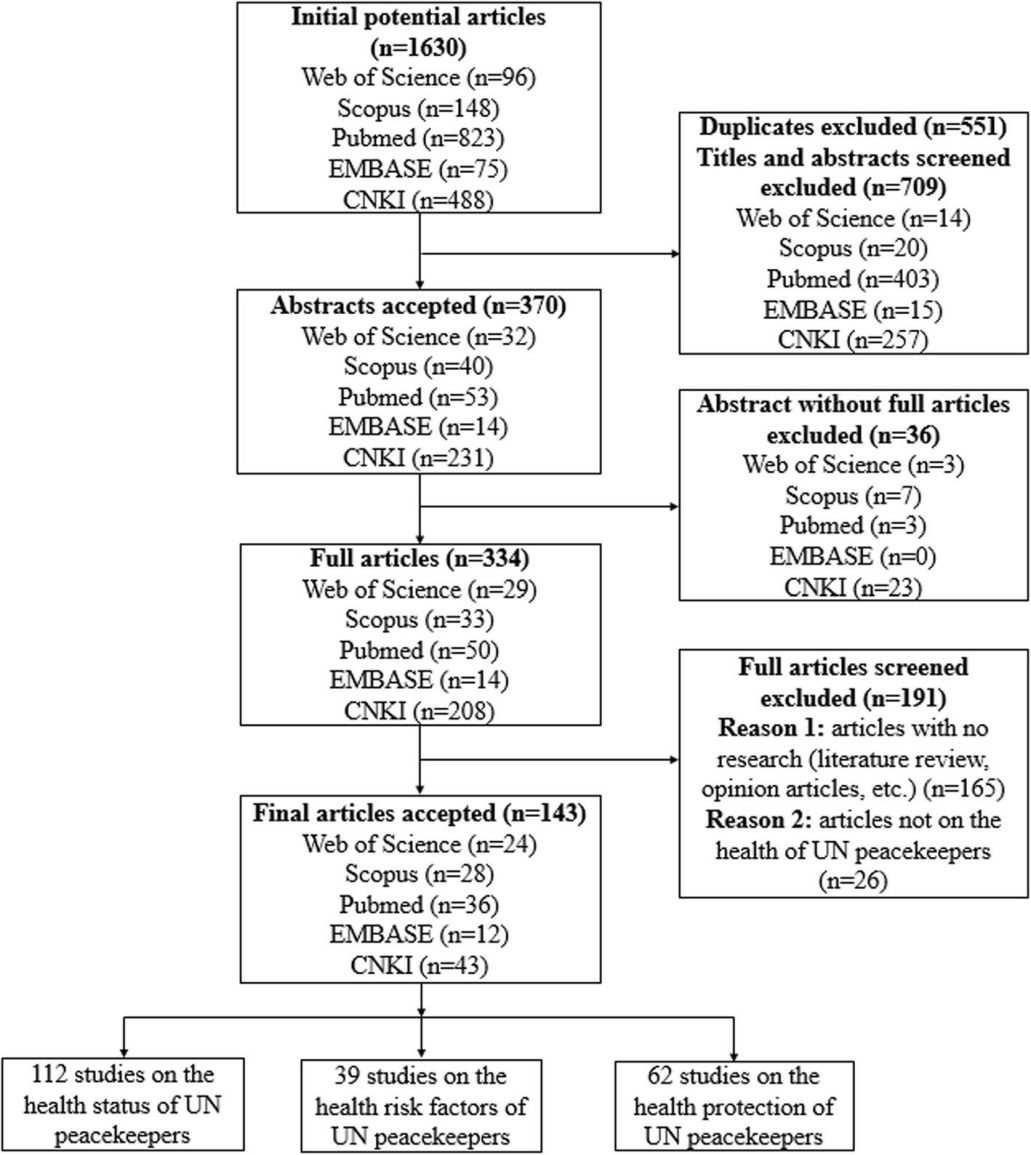
PRISMA flow chart to show the study selection process. This figure presents the process of selecting research on the health of UN peacekeepers

A total of 1630 records from our initial search in the databases were eligible for title and abstract screening. After removing duplicates (*n* = 551), 1079 studies were eligible for title and abstract screening. After title and abstract screening, 709 articles were deemed irrelevant and excluded. Then 36 articles were excluded without full texts. After full articles screened, 143 articles were included in the final analysis. The selection process is shown in the PRISMA flow diagram (Fig. 1).

### Health status of UN peacekeepers

#### Mental health

Totally 47 articles reported mental health problems, including PTSD [13–15, 17–24, 26–37], stress [38–45], anxiety and depression [35, 39, 46–60, 62, 63], and psychiatric disorders [13, 33, 46].

1. PTSD: Two studies reported the high prevalence of PTSD and symptoms among UN peacekeepers [13, 14], ranging from about 5to 20% in a short time after service. Three studies mentioned UN peacekeepers’ exposure to stressful events during the missions caused PTSD symptoms after deployment, based on epidemiological study, cross-sectional survey and multi-state models [31, 36, 37]. Notably, one study mentioned that 23 Indian peacekeepers deployed during the study period (2011–2015) reported no PTSD case, which might because that training and experience on counterinsurgency operations and stress busting measures received by Indian soldiers served them well during UN peacekeeping [35]. Eight studies mentioned predictors, measures and models for PTSD among peacekeepers such as the Self Rating Inventory for PTSD, to assess the broad range of symptoms and the severity of posttraumatic morbidity [20, 23, 24, 26, 28, 29, 33]. Two studies identified a small but significant percentage of peacekeepers with PTSD (8%), and explored the significant association between PTSD symptom severity and increased reports of stressor exposure among U.S. peacekeers in Somalia [21, 22]. Two studies explored Dutch peacekeepers’ PTSD in Cambodia and Yugoslavia through the trauma checklist and MMPI [15, 16]. One study pointed out the association between PTSD and suicidal ideation (6%) among Norwegian peacekeepers [25].
2. Stress: One study found peacekeepers with higher trait anxiety showed a reduced heart rate increase to the acute psychological stress compared to those with lower trait anxiety [44]. Two studies mentioned that UN peacekeepers from an economically poor environment, large families, and a history of unemployment had the high levels of stress due to unfamiliar environment and demanding work [38, 39]. One study investigated the relationship between stress symptoms and perceived organizational support among 1039 peacekeepers in Kosovo [43], and another study indicated the mechanisms of stress effects on cognitive function and health status among 72 Bulgarian peacekeepers in Kosovo. One study explored the relationship between the meaningfulness of work, personality hardiness, and their deriving benefits from stressful experiences among U.S. peacekeepers in Bosnia [42]. One study explored stress among Pakistani peacekeepers (4%) and highlighted the fact that they were resilient enough to handle the challenges of international environment [45]. Another study also explored the incident stress and suggested effective occupational health programs among U.S. peacekeepers [41].
3. Anxiety and depression: One study identified association between combat exposure and mental disorders including anxiety and depression [48]. Three studies mentioned the possibility of co-existing depression and anxiety symptoms in patients with PTSD [20, 33, 34]. Four studies used scales such as Self-Rating Depression Scale and Self-Rating Anxiety Scale to analyze the mental health status of peacekeepers before, during and after deployment, and identified around 6.9% prevalence of depression and around 7.9% prevalence of anxiety [53, 58, 59, 62]. Four studies mentioned the use of the Chinese Military Mental Health Scale and the Military Maladaptive Scale to measure Chinese peacekeepers’ mental health status, and to find the correlation between their personality characteristics and their mental health levels [50, 52, 60, 61]. Six studies used the Symptom Checklist and the Eysenck Personality Questionnaire to evaluate the mental health status of peacekeepers, and found the mental health status of peacekeepers in the medical team was better than those in the transportation team [51, 54–57, 108].
4. Psychiatric disorders: Three studies reported on psychiatric illness and disorders such as panic disorder and substance abuse of UN peacekeepers, associated with suicidal ideation after experiencing frequent panic attacks, among which Australian peacekeepers in Somalia had much higher morbidity than other UN soldiers [13, 33, 46].

#### Infectious diseases

Totally 14 articles reported infectious diseases, including HIV/AIDS [64–69], malaria and cholera [64, 67, 70–84], Hepatitis E virus (HEV) and Hepatitis A virus (HAV) [85–87], and other infectious diseases [88, 90, 91, 132, 134].

1. HIV/AIDS: Three studies pointed out that major efforts to prevent HIV among peacekeepers through HIV policies, which prevented HIV-positive persons from recruitment, enlistment, and deployment but implementation procedures of the policies differ greatly across different militaries [65, 68, 69]. One study investigated 3 cases of HIV/AIDS among Chinese peacekeepers in the Democratic Republic of the Congo [64]. Two studies focused on exploring Chinese peacekeepers’ knowledge on AIDS, one on the use of knowledge, belief and behavior questionnaire recommended by WHO among 528 peacekeepers in Liberia before and after health education [66], and another on 99.2% (264/266) of peacekeepers’ understanding AIDS transmission routes in Sudan [67].
2. Malaria and cholera: Four studies reported the use of chemoprophylaxis agianst cholera and malaria, anti-malarial therapy, screening and vaccination [76–78, 81]. Seven studies reported imported cases and high rates of malaria transmission and intended to characterize the potential causes of malaria outbreak among peacekeepers [70, 76, 79, 80, 82, 83]. Six studies mentioned the care for Chinese peacekeepers against malaria and analyzed the incidence characteristics of malaria and cholera among Chinese peacekeepers [64, 71–75]. One study reported a case of falciparum malaria complicated with bronchopneumoniain the Democratic Republic of the Congo [84], and another study researched on malaria and cholera prevention knowledge among Chinese peacekeepers in Sudan [67].
3. Hepatitis E virus (HEV) and Hepatitis A virus (HAV):: Two studies receptively explored Bangladeshi peacekeepers’ infection of HEV prior to deployment and determined the prevalence of HEV infection among peacekeepers from the United Nations Mission in Haiti and Haitian civilians [85, 86]. One study explored a 41-year-old Chinese peacekeeper who manifested fever, jaundice and coagulation dysfunction, and received treatment of severe acute HAV infection in South Sudan [87].
4. Other infectious diseases: Two studies reported dengue fever cases among peacekeepers and outlined measures to prevent local transmission [88, 132]. One study mentioned the prompt ribavirin treatment of clinically suspected Lassa cases in Sierra Leone [90]. One study mentioned the Ebola assessment and care provided in the Chinese Ebola Treatment Unit by Chinese peacekeepers [91]. One study identified that Chinese peacekeepers who were repeatedly deployed to high-risk areas of yellow fever might not be seriously affected by yellow fever virus [89].

#### Other health problems

Totally 39 articles reported other health problems, including oral, skin, gastrointestinal, musculoskeletal, respiratory, urinary, ophthalmic, otolaryngologic and gynecological diseases [64, 71, 80, 82, 94–100, 107–109, 112, 138, 141, 147], skin diseases [101, 104, 110], leukemia [102, 103, 111], and others [92, 93, 105, 106, 113, 114, 116, 117].

1. Oral, skin, gastrointestinal, musculoskeletal, respiratory, urinary, ophthalmic, otolaryngologic and gynecological diseases: Fourteen studies conducted comprehensive and retrospective analysis on disease characteristics of Chinese peacekeepers, including otorhinolaryngology diseases, respiratory system diseases, fracture, digestive, endocrine and metabolic diseases [64, 71, 80, 82, 95, 96, 98, 100, 107, 108, 112, 138, 141, 147]. Two studies mentioned the musculoskeletal pain among Swedish peacekeepers in Afghanistan and Canadian peacekeepers in Bosnia between 2000 and 2004 were characterized [94, 99]. One study pointed out health hazards including arthropod-borne, food and water-borne, respiratory tract diseases, enzootic diseases, battle injuries, and non-battle injuries when conducting the UN peacekeeping missions in the Middle East (Lebanon, the Golan Heights) [97]. One study described the combat-related injuries cured by the UN second level medical treatment facility in Mali [109]. One study comprehensively analyzed the diagnosis and treatment of ophthalmology in the UN second level hospital in the Democratic Republic of the Congo [139].
2. Skin diseases: Two studies explored Chinese peacekeepers’ injury characteristics, treatment and prognosis of skin and soft tissue defects in second-level peacekeeping hospitals of Mali and Lebanon [101, 110]. One study outlined the dermatological profiles of international peacekeepers in UN second level hospital to retrospectively assess the disease patterns and made comparisons with previous skin disease reports among peacekeepers [104].
3. Leukemia: Two studies mentioned the aroused alert on the exposure to depleted uranium associated with leukemia among European peacekeepers who served in Iraq and the Balkans [102, 103]. One study compared the post-war cause-specific mortality of 53,320 veterans who deployed to Bosnia/Kosovo between 1996 and 2002 to that of 117,267 veterans who were not, and found increased risks of disease related mortality among U.S. peacekeepers [111].
4. Others: One study monitored serologic evidence of exposure to diseases that caused acute febrile illness among Mongolian peacekeepers in South Sudan [106]. One study was conducted to investigate fatal accidents in Norwegian former peacekeepers, and found the need for preventive measures at reducing the risk of accidental death [93]. One study explored peacekeepers’ hydration status and cardiac autonomic modulation in Haiti, and found that an operational peacekeeper patrol promoted both dehydration and an imbalance in the autonomic modulation of soldiers’ heart rate [117]. One study described patterns of homicidal violence among peacekeepers and suggested possible reasons for the attacks [116]. One study explored the increasing proportion of acute acute appendicitis among 462 inpatients in a peacekeeping secondary hospital of Kinshasa from January 2017 to December 2019 [114]. One study pointed out the importance of effective surveillance and good microbiology laboratory support towards immunization against rubella during deployments [92]. One study reported on ultrasound used in abdominal, superficial, obstetrics and gynecology, and cardiovascular to check peacekeepers’ health in the Democratic Republic of the Congo [113]. One study demonstrated that the most serious health problems occurring in the group of Polish soldiers in the given period included digestive tract diseases (12.9%) and non-battle injuries (9.2%) [115]. One study found conflict exposure was positively correlated with increased risk of mortality from non-neoplastic diseases among Norwegian peacekeepers in Lebanon [105].

### Health risk factors of UN peacekeepers

#### Natural environmental factors

Totally 8 articles reported on natural environmental factors as health risk factors of UN peacekeepers in multiple troop-contributing and host countries [62, 64, 67, 97, 114, 118–120].

Two studies reported the tough and harsh natural environment in host countries, including mold, fungi, and dust, wind, sand, and mountain conditions, which may cause health problems such as heat injuries and low temperature injuries among peacekeepers [62, 67, 97, 118, 120]. Two studies reported seasonal distribution such as hot climate, sufficient rainfall, dense vegetation as health risk factors of UN peacekeepers in host countries [64, 114]. One study mentioned most host countries were located in tropical areas, suitable for the survival of many pathogenic bacteria [119].

#### Social environmental factors

Totally 24 articles reported on social environmental factors as health risk factors of UN peacekeepers in multiple troop-contributing and host countries [17, 35, 38, 39, 43, 47, 49, 52, 53, 62, 64, 80, 104, 110, 112, 118, 121–127, 150].

Seven studies reported peacekeepers’ economic conditions and family relationships such as their marriage status are associated with their health status [35, 38, 39, 121–123, 150]. Ten studies found that previous unemployment, complex war environment, closed camp environment, lack of meaningfulness in stressful mission, inherent work dangers, and differences in culture made peacekeepers actively adapt to social environments, otherwise led to health problems during deployment [17, 38, 47, 49, 62, 104, 110, 120, 125]. Five studies pointed out the importance of social and organizational support, such as providing collective pre-deployment training and improving local health resources, to ensure peacekeepers’ health [43, 52, 64, 80, 126]. Two studies mentioned traffic accidents, munitions and explosives fracture compound injuries among peacekeepers [112, 118]. One study investigated factors associated with PTSD symptoms, including lower level of education, being single, and more traumatic situations during deployment [124]. One study mentioned that peacekeepers’ perception of their spouses’ support during the deployment had a positive impact on work-to-family enrichment, which mediated their health perception and general satisfaction with life [127].

#### Psychological factors

Totally 13 articles reported on psychological factors as health risk factors of UN peacekeepers in multiple troop-contributing and host countries [15, 24, 37, 42, 47, 49, 53, 62, 104, 121, 125, 126, 128].

Four studies mentioned that peacekeepers often suffered from distress because of missing hometown and disharmony in interpersonal relationships, which led to long-term mental fatigue [53, 62, 104, 125]. Four studies mentioned peacekeepers’ personal barriers to disclose experiences such as traumatic events [15, 24, 37, 42]. Three studies suggested peacekeepers to actively cope with mental health distress, have various mental health activities, and build confidence and resilience throughout the deployment [47, 49, 126]. Two studies highlighted peacekeepers’ mental health needs and barriers including peacekeepers’ feeling of more powerless and threatening, the idea that the mission had become meaningless, and having had no control over the situation [121, 124]. One study mentioned that when peacekeepers’ hardiness were high, their psychological distress became low [128]. One study found no association between psychosocial factors (military peace force stressors, clinical stress, anxiety and depression) and blood T lymphocyte among Brazilian peacekeepers in Haiti [129].

#### Behavioral lifestyle factors

Totally 9 articles reported on behavioral lifestyle factors as health risk factors of UN peacekeepers in multiple troop-contributing and host countries [14, 30, 52, 71, 93, 104, 114, 125, 131].

Five studies showed peacekeepers’ misuse of alcohol and lack of drinking water and having food due to uncertainty during the deployment led to their health problems [14, 30, 71, 93, 114]. Three studies showed peacekeepers’ group living lifestyle with different cultural backgrounds, religious beliefs, and customs require them to improve their adaptability to ensure group work proficiency [52, 104, 125]. One study explored severity PTSD symptoms impacted by exposure to combat directly and indirectly through fear and sexual harassment among U.S. peacekeepers in Somalia [130]. One study explored understanding the complex association among peacekeepers’ PTSD, coping style, and resilience by focusing on the experiences of Chinese peacekeepers in South Sudan [131].

#### Biological factors

Totally 3 articles reported on biological factors as health risk factors of UN peacekeepers in multiple troop-contributing and host countries [53, 64, 110].

Three studies reflected on the causes of infectious disease such as malaria, including poor sanitary conditions and the high prevalence of patients and carriers [53, 64, 110].

#### Health service factors

Totally 3 articles reported on health service factors as health risk factors of UN peacekeepers in multiple troop-contributing and host countries [53, 64, 110].

Two studies mentioned health services included health and disease prevention tasks and the preparation of medical materials such as antimicrobial chemoprophylaxis to avoid infections [64, 110]. One study mentioned the provision of beneficial mental health care resulted in the improvement of peacekeepers’ mentality [53].

### Health protection of UN peacekeepers

#### Disease prevention measures

Totally 22 articles reported on disease prevention measures as health protection of UN peacekeepers in multiple troop-contributing and host countries [64–68, 70, 73, 74, 76, 78–80, 84, 88, 92, 101, 107, 114, 132–135].

Eight studies mentioned the enforcement of preventive medicine such as chemoprophylaxis and antiallergic drugs, immunization, disinfection, screening [67, 74, 76, 78, 84, 88, 92, 101]. Nine studies emphasized the health education on disease prevention as health protection of UN peacekeepers, such as HIV/AIDS knowledge, malaria chemoprophylaxis and medical examinations [64, 66, 68, 73, 79, 80, 107, 114, 134]. Six studies pointed out the improvement of disease prevention system and policy, including early notification and isolation of cases, diagnosis and management of infectious diseases, malaria protection policy, improved detection systems for mental health problems and individual interventions [65, 70, 107, 132, 133, 135].

#### Medical and health measures

Totally 22 articles reported on medical and health measures as health protection of UN peacekeepers in multiple troop-contributing and host countries [20, 29, 64, 72, 75, 79, 80, 87, 91, 92, 94, 95, 101, 107, 112, 114, 136–141].

Nine studies mentioned medical and health measures included vaccines, dietary nutrition, hospital treatment such as specialized PTSD treatment and anti-malarial treatment, physiotherapy services, isolation measures, drug preparations [20, 72, 75, 80, 87, 91, 94, 95, 137]. Nine studies pointed out it was important to reinforce coordination with overseas healthcare services, equip specialized professionals and complex equipment, optimize the diet structure, standardize medical surveillance, improve treatment knowledge and plan [79, 92, 101, 107, 112, 114, 136, 138, 139]. One study listed specific examples of a thorough evaluation of severity of subclusters to deal with peacekeepers’ health problems [29]. Three studies emphasized food hygiene, disinfection of drinking water, and frequent hand washing as medical and health measures for peacekeepers’ health protection [64, 140, 141].

#### Psychosocial measures

Totally 29 articles reported on psychosocial measures as health protection of UN peacekeepers in multiple troop-contributing and host countries [13, 14, 29, 31, 35, 38–40, 43, 44, 54, 56, 57, 67, 80, 82, 95, 107, 116, 122, 138, 140, 142, 144–149].

One study explored the impact of a powerful TV drama on the psychological health of U.K. peacekeepers compared with other military personnel [31]. Sixteen studies reported psychosocial measures included changing regulations, providing pre-deployment training and guidance, mental health education through lectures and broadcasts, organizational support programs, psychological counseling and intervention [13, 14, 35, 39, 43, 54, 56, 82, 107, 140, 142, 144–147, 149]. Six studies pointed out there were approaches to stress management and assessment, and peacekeepers were taught to express emotions with teammates and families [29, 38, 40, 44, 116, 122]. Four studies showed the medical standard working and environmental management procedures, to increase peacekeepers’ disease awareness and strengthen medical protection [67, 80, 95, 138]. Two studies showed the use of psychosocial measures such as the Tachikawa Resilience Scale to know about peacekeepers’ coping styles during the deployment [57, 148]. One study investigated perceived psychological needs and found that they did not require formalised interventions due to already accessing formalised support mechanisms, but additional social support from peers and family were needed among U.K. peacekeepers [143].

#### Diplomatic measures

Totally 2 articles reported on diplomatic measures as health protection of UN peacekeepers in multiple troop-contributing and host countries [69, 83].

One study mentioned the malaria elimination campaigns shared by UN peacekeepers from Sri Lanka, which had malaria eradication since 2012 [83]. One study pointed out the incoherent UN policy did not empower the military to exclude HIV positives, which could be seemed as a major factor to find HIV-positive peacekeepers, so better military HIV policies could be used to ensure uniform standards in the teams [69].

## Discussion

### Main findings

This scoping review demonstrated that the existing research primarily concentrated on the general health status, health risk factors and health protection of UN peacekeepers in multiple troop-contributing and host countries. Many research explored the health status of UN peacekeepers in Africa deployed from mainly the U.S., Canada, U.K., China, Australia and Norway, and reported mainly on mental health problems and infectious diseases. The current analysis on the health risk factors of UN peacekeepers mainly focused on host countries, and covered natural, social, psychological, behavioral, biological and health service perspectives. The current exploration on health protection of UN peacekeepers was mainly based on previous experience of UN peacekeepers, specifically on disease prevention, medical, psychosocial, and diplomatic measures. However, there was a lack of strategic explorations on complex health risk factors of UN peacekeepers and comprehensive strategies in health protection before, during and after the deployment.

This scoping review found the current research on the health status of UN peacekeepers mainly covered mental health, infectious diseases and a diversity of other diseases. Most peacekeepers suffered from PTSD after deployment [21, 26], experienced stress during deployment [41, 45], reported anxiety and depression after mental health scale tests [52, 61], and a few serious cases experienced psychiatric illness and suicidal ideation [13, 33]. The majority of articles covered the general mental health status from multination in peacekeeping, with U.S., U.K., Canada, Australia, and China reporting more than other countries on mental health problems among peacekeepers. HIV/AIDS, malaria and cholera were identified and researched by multination including Nigeria, China, Egypt, Sri Lanka, and Bangladesh which sent peacekeepers to Haiti, the Democratic Republic of the Congo, Sudan, Liberia, and South Sudan. Other infectious diseases such as Dengue, Lassa and Yellow fever were identified by a few troop-contributing countries and reported cases for further investigation. Other health problems such as oral, skin, gastrointestinal, musculoskeletal, and respiratory diseases mainly reported by Chinese researchers, according to the diagnosis and treatment report in the Chinese secondary hospitals [104, 109, 139, 151]. Leukemia and cancer were explored mainly by countries such as U.S. and Italy, while dehydration and non-battle injuries were explored by countries such as Brazil and Poland [102, 103, 115, 117].

Global health is the study of health problems, health issues, and health concerns across national boundaries, which could influence the health of people through environment and experience of countries around the world [152]. Global health problems have cross-border characteristics, whose impact can be transferred to other countries. Our previous research on the Chinese medical team during the Ebola pandemic in Liberia showed that peacekeepers faced the challenges of Ebola as well as other infectious diseases (HIV, malaria, and tuberculosis) and psychological stressors (fear and anxiety) [153]. And the COVID-19 outbreak has again posed threats to peacekeepers’ health problems and led to severe diseases acorss borders [154]. A review by Shigemyra et al. also identified the association between UN peacekeepers’ exposure to multiple events and the further development of their health problems after deployment [155]. Drabick and Kunkel listed examples of UN Peacekeepers contributing to disease spreading, such as Hepatitis E Infection and the Cholera in Haiti [81, 85]. The health problems of UN peacekeepers are complicated, and whose impacts are cross-border.

This scoping review found that social environmental factors were explored the most among other health risk factors. Peacekeepers’ economic conditions, family relationships, previous employment, war environment, cultural differences, and education level seemed to be social environmental factors associated with their health status, reported by a range of countries such as Norway, U.S., U.K., and China in some peacekeeping areas such as Bosnia, Lebanon and Kosovo [38, 62, 121, 124]. A number of troop-contributing countries such as Australia and Netherland also explored psychological factors as health risk factors of UN peacekeepers in host countries such as Lebanon and Haiti. Due to missing hometown, disharmony in interpersonal relationships, and personal barriers to disclose experiences could lead to peacekeepers’ psychological distress such as anxiety and depression during and after deployment [49, 104, 124, 129]. Natural environmental factors and behavioral lifestyle factors were also seemed to be important health risk factors of UN peacekeepers multiple troop-contributing such as Finland and China, and host countries such as the Democratic Republic of Congo and Somalia. Tough natural environment, hot climate, sufficient rainfall and dense vegetation in host countries may cause health problems such as heat injuries and pathogenic bacteria [97, 114, 119], while misuse of alcohol, lack of drinking water and food, group living lifestyle, fear and sexual harassment could lead to their health problems during the deployment [14, 93, 130]. Only a small number of articles reported on health service factors and biological factors as health risk factors of UN peacekeepers from China to Mali, Lebanon, and the Democratic Republic of the Congo. Poor sanitary conditions led to infectious disease such as malaria, health and disease prevention services could avoid infections and mental health care could improve peacekeepers’ mentality [53, 64, 110, 156].

The health risk factors of UN peacekeepers are complicated and across different countries. Our previous study on the Chinese medical team during the Ebola pandemic in Liberia pointed out that the resource-limited working environment and the underdeveloped local public health system could be health risk factors among UN peacekeepers [153]. The limited access to health facilities, the lack of vaccinations, and the insufficient use of personal protective equipment such as facial masks and personal hygiene products could be health risk factors among UN peacekeepers during the COVID-19 pandemic [154]. The completion of UN peacekeeping missions required the cooperation between host countries and troop-contributing countries, but this scoping review found that the analysis on the health risk factors of UN peacekeepers was mainly about host countries. Mehlum and Korzeniewski identified different health risk factors among UN peacekeepers, including their tough living conditions and unhealthy lifestyles [14, 97]. According to the World Health Organization, the Social Determinants of Health (SDH) are the conditions in which people are born, grow, work, live, and age, and the wider set of forces and systems shaping the conditions of their daily life [157]. The SDH of UN peacekeepers not only included the health risk factors in host countries, but also involved health risk factors across different countries.

This scoping review found that disease prevention measures, medical and health measures, and psychosocial measures were the main health protection for UN peacekeepers. Psychosocial measures included pre-deployment training, mental health education, organizational support programs, psychological counseling and intervention, stress management and assessment, and social support from peers and family, to increase peacekeepers’ disease awareness and strengthen health protection among peacekeepers from countries such as U.S., U.K., China, Ireland, Bulgaria, etc. who deployed to countries such as Yugoslavia and Liberia [67, 80, 138, 143, 158]. Disease prevention measures included the enforcement of preventive medicine, the health education on disease prevention, the improvement of disease prevention system and policy reported by troop-contributing countries such as Nigeria and U.K. to host countries such as Bosnia–Herzegovina and South Sudan [65, 76, 88, 107, 133]. Medical and health measures included reinforcing vaccines, improving hospital treatment, optimizing the diet structure, standardizing medical surveillance, disinfection of drinking water, and frequent hand washing for peacekeepers’ health protection from troop-contributing countries such as China and Canada to host countries such as Lebanon and Mali [20, 29, 79, 141]. Only Guerra and Assan explored the use of diplomatic measures such as the malaria elimination campaigns and the reinforcement of HIV policy in the UN, to ensure the diplomatic impact on peacekeepers’ health protection [69, 83].

There are existing programs, policies, regulations, common practices, and rules around the health of UN peacekeepers to prevent the spread of diseases. The Security Council has a peace operation resolution by setting out missions’ mandates and monitoring the work of UN peacekeepers through periodic reports [159]. The General Assembly monitors the performance of UN Peacekeeping through the Special Committee on Peacekeeping Operations established in 1965, and discusses on matters regarding the health protection of UN peacekeepers through the *Uniting for Peace* resolution established in 1950 [160]. The UN Department of Operational Support has a six-year strategy on operating at minimum risk to UN peacekeepers, troop-contributing and host population, societies and ecosystems, by making on-site risk assessments and promptly implementing health actions [161]. The UN Peacekeeping Capability Readiness System aims to establish a dynamic interaction between the UN Headquarters and Member States to strengthen UN peacekeepers’ health readiness and timely deployment during the process [162]. However, measures demontrsated in this scoping review and existing programs in the UN were taken mainly based on previous experience and lessons, with a lack of theoretical guidance from global health perspectives.

### Implications for practice and research

As mentioned above, since health problems of UN peacekeepers are global health issues, health protection of UN peacekeepers could be taken from global health perspectives. Given multiple health risk factors of UN peacekeepers, more formative and implementation research are needed to explore potential strategies on the health protection among peacekeepers.

#### Multi-phases

The health protection of UN peacekeepers can be divided into three phases. Firstly, before deployment, troop-contributing countries could improve UN peacekeepers’ health protection knowledge and take preventive measures such as physical examination, health education, psychological counseling, and vaccination [163, 164]. Secondly, during the deployment, it is necessary to establish effective health systems that can provide sufficient medical and health services for UN peacekeepers, help them develop healthy life habits, and enhance their health protection skills [138, 165, 166]. Thirdly, after the deployment, it is necessary to pay attention to UN peacekeepers’ health conditions and infection risks among wide populations, so it is recommended to take preventive measures for transportation and quarantine when they arrive home [164].

#### Multi-disciplines

The health protection of UN peacekeepers requires support from multiple disciplines including public health, medicine, politics, health diplomacy, and others. Firstly, public health. The health protection of UN peacekeepers requires insights from the public health discipline to prevent infections in large groups [68, 87]. Lowicki-Zucca et al. mentioned UN peacekeepers posed public health threats to troop-contributing countries, which need public health experts’ solutions and actions on health education and prevention measures [68]. Secondly, medicine. Experts from clinical and preventive medicine backgrounds could provide UN peacekeepers with medical treatment [154, 167]. Lewnard et al. pointed out that screening and vaccination were effective strategies to prevent cholera introduction during large-scale UN peacekeeping deployments such as in the 2010 Haiti outbreak [78], so clinical and preventive medicine are needed in disease prevention and control. Thirdly, politics. Since global health is dedicated to improving health equity for all human beings and integrating health protection into policies, political bodies could cooperate and take political actions such as implementing regulations and laws to protect UN peacekeepers’ health [69, 154]. Despite political actions taken by the UN on HIV transmission among UN peacekeepers, military HIV policies could be facilitated to ensure uniform standards, interpretation, and implementation to guide UN peacekeepers’ health behaviors [69]. Fourthly, diplomacy. It is critical for governments and organizations to take humanitarian measures in health diplomacy from regional, bilateral, and multilateral perspectives [126, 167]. Stakeholders involved in UN peacekeeping could take actions to urge political unity, build trust among public institutions, and ensure health equities among UN peacekeepers [167]. Fifthly, other disciplines such as logistics management and sociology could be used in their health protection measures [57, 154]. For instance, the logistic support of medical drugs and equipment could have critical influence on the health protection of UN peacekeepers against parasites and epidemics caused by viruses and bacteria [154].

#### Multi-levels

The UN peacekeeping is a global mission, which requires global health protection from multi-levels, including the UN, host countries, troop-contributing countries, the UN peacekeeping team, and UN peacekeepers. Firstly, the UN could establish graded treatment institutions and sufficient mechanisms, to ensure health protection and epidemic prevention among peacekeepers. Secondly, host countries and troop-contributing countries could take responsibilities of training peacekeepers [94]. Thirdly, UN peacekeeping teams could cooperate and provide health protection for peacekeepers, by including the first-level medical treatment team deployed by most peacekeeping units, and the second-level medical treatment team held by medical professionals from various countries or jointly organized by the surrounding hospitals [109, 135]. Fourthly, UN peacekeepers themselves could take initiatives of improving health literacy by learning health protection knowledge to ensure the progress of peacekeeping missions [41, 109].

### Limitations

There are several limitations in this review. Firstly, most measures mentioned in articles of this scoping review were not tested by intervention studies, which could be further practiced and researched. Secondly, we added languages other than English or Chinese as additional filters to search on databases, and found no more articles in other languages on this topic.

## Conclusions

This scoping review synthesized current studies on UN peacekeepers’ health. This review informed that current research topics on UN peacekeepers’ health mainly covered health problems and health risk factors, with a lack of comprehensive health protection measures. UN peacekeepers’ health problems are typical global health issues, with complicated and cross-border health risk factors. However, the review showed that the current health protection of UN peacekeepers was mainly based on previous experience of UN peacekeepers. Therefore, more comprehensive strategies could be taken from global health perspectives, including multi-phases, multi-disciplines, and multi-levels. The future practice and research on the health of UN peacekeepers is of great significance, in terms of improving the health protection of peacekeepers and consolidating the peacekeeping effectiveness.

### Supplementary Information

**Additional file 1.** Search Strategy

## Data Availability

Not applicable.

## Abbreviations

UN: United nations
PRISMA: Preferred reporting items for systematic reviews and meta-analyses
CNKI: China national knowledge infrastructure
AIDS: Acquired immune deficiency syndrome
HIV: Human immunodeficiency virus
HEV: Hepatitis E virus
HAV: Hepatitis A virus
UNAIDS: Joint united nations program on HIV/AIDS
PTSD: Post traumatic stress disorder
COVID-19: Corona virus disease 2019
SDH: Social determinants of health
