# Correction: UN peacekeeper health and risk factors --- a systematic scoping review

**DOI:** 10.1186/s41256-024-00355-0

**Published:** 2024-04-24

**Authors:** Quan Yuan, Yong Chen, Shili Liu, Qingning Huang, Miaomiao Liao, Jiani Zhou, Zhaogang Li, Ying Li

**Affiliations:** Department of Social Medicine and Health Service Management, Army Medical University (Third Military Medical University), Chongqing, China

**Correction: Glob Health Res Policy 9, 13 (2024)**

**https://doi.org/10.1186/s41256-024-00351-4**

Following publication of the original article [1], it is noticed that the funding number is incorrect. The correct funding information is below:

This study was funded by the project (cstc2015shmszx120070). The funder had no role in the study design, data collection, and analysis, decision to publish, or manuscript preparation.

The original article [1] has been corrected.
