# Supplementary material for: UN peacekeeper health and risk factors --- a systematic scoping review
Source: Glob Health Res Policy. 2024 Apr 10;9:13. doi: 10.1186/s41256-024-00351-4 (PMC11005225; doi:10.1186/s41256-024-00351-4)
Supplement: Supplementary file 1 — Additional file 1. Search Strategy [file 41256_2024_351_MOESM1_ESM.docx]

**Search Strategy**

**Database: Scopus**

| 1. ( TITLE-ABS-KEY ( peacekeepers ) AND TITLE-ABS-KEY ( health OR disease OR infection OR prevalence ) ) AND ( LIMIT-TO ( DOCTYPE , "ar" ) ) | 133 |
| --- | --- |
| 1. ( TITLE-ABS-KEY ( peacekeepers ) AND TITLE-ABS-KEY ( health AND risk OR risk AND factor OR health AND risk AND factor ) ) AND ( LIMIT-TO ( DOCTYPE , "ar" ) ) | 10 |
| 1. ( TITLE-ABS-KEY ( peacekeepers ) AND TITLE-ABS-KEY ( health AND protection OR health AND measure OR health AND prevention OR health AND policy ) ) AND ( LIMIT-TO ( DOCTYPE , "ar" ) ) | 5 |

**Database: Web of Science**

| 1.(TS=( (UN peacekeepers OR United Nations peacekeepers))) AND TS=((health OR disease OR infection OR prevalence)) | 58 |
| --- | --- |
| 1. ((TS=( (UN peacekeeper OR United Nations peacekeeper))) AND TS=(health)) AND TS=( (risk factor OR risk)) | 18 |
| 1. ((TS=( (UN peacekeepers OR United Nations peacekeepers))) AND TS=(health)) AND TS=( (protection OR measure OR prevention OR policy OR control)) | 20 |

**Database: PubMed**

| 1. (((((UN peacekeeper) OR United Nations peacekeeper) OR peacekeeper) AND ((((health) OR disease) OR infection) OR prevalence))) | 410 |
| --- | --- |
| 2. (((((UN peacekeeper) OR United Nations peacekeeper) OR peacekeeper) AND (((health risk factor) OR health risk) OR health factor)) | 126 |
| 3. (((((UN peacekeeper) OR United Nations peacekeeper) OR peacekeeper) AND ((((((protection) OR measure) OR prevention) OR policy) OR control)) | 287 |

**Database: Embase**

| 1. ('peacekeeper' OR 'united nations peacekeeper' OR 'un peacekeeper') AND ('health'/mj/exp OR 'health'/exp OR 'health' OR 'disease'/mj/exp OR 'disease'/exp OR 'disease' OR 'infection'/mj/exp OR 'infection'/exp OR 'infection' OR 'prevalence'/mj/exp OR 'prevalence'/exp OR 'prevalence') | 48 |
| --- | --- |
| 1. ('peacekeeper' OR 'united nations peacekeeper' OR 'un peacekeeper') AND ('health risk'/exp OR 'risk factor'/exp OR 'health risk factor') | 4 |
| 1. ('peacekeeper' OR 'united nations peacekeeper' OR 'un peacekeeper') AND ('health'/exp OR 'health' OR 'disease'/exp OR 'disease') AND ('protection'/exp OR 'protection' OR 'measure' OR 'policy'/exp OR 'policy' OR 'prevention'/exp OR 'prevention' OR 'control'/exp OR 'control') | 23 |

**Database: CNKI**

| 1. (SU = 'Peacekeeper') AND (SU = 'Disease' OR SU = 'Infection' OR SU = 'Health') | [311](https://www-webofscience-com.myaccess.library.utoronto.ca/wos/woscc/summary/6e21ad14-eea3-42f6-a395-3c41975a0c2c-2a1fe3d5/relevance/1) |
| --- | --- |
| 1. (SU = 'Peacekeeper') AND (SU = 'Health Factor' OR SU = 'Influencing Factor') | [30](https://www-webofscience-com.myaccess.library.utoronto.ca/wos/woscc/summary/b85ccb5b-1e26-484a-922c-991311a153c9-2a1fe2fe/relevance/1) |
| 1. (SU = 'Peacekeeper') AND (SU = 'Control' OR SU = 'Measures' OR SU = 'Prevention' OR SU = 'Strategy') | 147 |
